# Fine-scale dissection of the subdomains of polarity protein BASL in stomatal asymmetric cell division

**DOI:** 10.1093/jxb/erw274

**Published:** 2016-07-15

**Authors:** Ying Zhang, Dominique C. Bergmann, Juan Dong

**Affiliations:** ^1^Waksman Institute of Microbiology, Rutgers, The State University of New Jersey, Piscataway, NJ 08854, USA; ^2^Department of Biology, 371 Serra Mall, Stanford University, Stanford, CA 94305–5020, USA; ^3^HHMI, Stanford University, Stanford, CA 94305–5020, USA; ^4^Department of Plant Biology and Pathology, Rutgers, The State University of New Jersey, Piscataway, NJ 08901, USA

**Keywords:** *Arabidopsis*, asymmetric cell division, cell polarity, polarity protein, stomatal development, subcellular localization.

## Abstract

Cell polarity is a prerequisite for asymmetric cell divisions (ACDs) that generate cell type diversity during development of multicellular organisms. In *Arabidopsis*, stomatal lineage ACDs are regulated by the plant-specific protein BREAKING OF ASYMMETRY IN THE STOMATAL LINEAGE (BASL). BASL exhibits dynamic subcellular localization, accumulating initially in the nucleus, but then additionally in a highly polarized crescent at the cell cortex before division. BASL polarization requires a phosphorylation-mediated activation process, but how this is achieved remains unknown. In this study, we performed a fine-scale dissection of BASL protein subdomains and elucidated a nuclear localization sequence for nuclear import and a critical FxFP motif for cortical polarity formation, respectively. Artificially tethering BASL subdomains to the plasma membrane suggests that novel protein partner/s might exist and bind to an internal region of BASL. In addition, we suspect the existence of a protein degradation mechanism associated with the amino terminal domain of BASL that accounts for restricting its predominant expression to the stomatal lineage cells of the epidermis. Taken together, our results revealed that BASL, through its distinct subdomains, integrates multiple regulatory inputs to provide a mechanism that promotes difference during stomatal lineage ACDs.

## Highlight

Analysis and manipulation of BASL subdomains reveal that this polarity protein integrates multiple regulatory inputs to promote difference during stomatal lineage asymmetric cell division.

## Introduction

Asymmetric cell division (ACD) plays a major role during the development of multicellular organisms (Horvitz and Herskowitz, 1992). Stem cells, in particular, divide asymmetrically to renew themselves while simultaneously generating cells that will differentiate. Plant development features continual post-embryonic generation of tissues and organs from a limited number of spatially restricted stem cells (De Smet and Beeckman, 2011). The molecular mechanisms underpinning plant ACDs have been a subject of active investigation in the past decades, but much of the work focused on fate asymmetry in daughter cells, rather than the initiation of polarity and asymmetry in the mother (Petricka *et al.*, 2009; De Smet and Beeckman, 2011; Kajala *et al.*, 2014).

A good system in which to investigate asymmetry is the stomatal lineage of *Arabidopsis*. Here, protodermal cells in the epidermis undergo ACDs to maintain the self-renewing precursors of the stomatal guard cells (Lau and Bergmann, 2012). Guard cells are used to modulate the opening and closing of stomata so that plants can acquire CO_2_ from the atmosphere while minimizing water loss. During stomatal development, the basic helix-loop-helix (bHLH) transcription factor SPEECHLESS (SPCH) together with its partner bHLHs, the SCREAM factors (SCRM/ICE1 and SCRM2), functions as a master regulator of the ACDs that initiate and maintain the lineage (MacAlister *et al.*, 2007; Kanaoka *et al.*, 2008). SPCH controls the expression of a wide range of downstream genes to specify stomata lineage cell fate, cell polarity, and cell-to-cell communication (Lau *et al.*, 2014).

Among SPCH targets are the polarly localized proteins BREAKING OF ASYMMETRY IN THE STOMATAL LINEAGE (BASL) (Dong *et al.*, 2009) and POLAR (Pillitteri *et al.*, 2011). The localization of these proteins is intriguing, but their roles as regulators of division asymmetry are much less understood, in part because the plant proteins show no similarity to the intrinsic polarity proteins (such as the PARs) conserved among animals (Knoblich, 2008; Inaba and Yamashita, 2012). In fact, BASL and POLAR encode plant-specific proteins of unknown biochemical functions. Recent studies, however, suggest that BASL functions as a scaffold protein by interacting with the Mitogen-activated protein kinase (MAPK) kinase kinase YODA (MAPKKK YDA) and the MAPKs MPK3 and MPK6, pivotal negative regulators of stomatal development (Bergmann *et al.*, 2004; Wang *et al.*, 2007). BASL ensures that MAPK activity is preferentially inherited by the larger daughter of an ACD; because SPCH is negatively regulated by MAPK activity, the differential MAPK signalling in two daughter cells leads to preservation of SPCH stem cell activity in one daughter but not the other (Zhang *et al.*, 2015).

Within an asymmetrically dividing cell, BASL is localized to two distinct subcellular compartments, the plasma membrane (PM) and the nucleus (Dong *et al.*, 2009). BASL’s subcellular localization is linked to cell behaviour, and the current model, based on observations of many BASL-expressing stomatal lineage cells, is that cells expressing BASL dually in the nucleus and at the PM undergo another ACD, whereas cells expressing nuclear-only or peripheral-only BASL differentiate into stomatal guard cells or epidermal pavement cells, respectively (Dong *et al.*, 2009). Therefore, proportioning BASL between the nucleus and the PM has phenotypic consequences and must be strictly controlled. In this study, we focused on intrinsic features of the BASL protein that lead to its localization. By detailed characterization of its subdomains and by manipulating their subcellular localization, we address several questions about this intriguing protein: (i) Which sequences in BASL regulate its nucleocytoplasmic trafficking? (ii) What motifs in BASL are critical for its ability to be polarized? and (iii) What domains might mediate protein–protein interactions critical for BASL function? Our results provide new insights into the basic properties of the novel polarity protein BASL and enlighten future research directions towards understanding the mechanisms for cell polarity and ACD in plants.

## Materials and methods

### Accession numbers

Sequence data can be found in the Arabidopsis Genome Initiative or GenBan/EMBL databases under the following accession numbers: *Arabidopsis thaliana* BASL (At5g60880), the proteins aligned with the conserved C-tail of BASL: rice (*Oryza sativa*; Os04g39240.1), maize (*Zea mays*; ZEAMMB73_558984), tomato (*Solanum lycopersicum*; XP_004239019) and medicago (*Medicago truncatula*; MTR_2g461550).

### Plant materials and growth conditions

*Arabidopsis thaliana* seeds were stratified for 1–2 days in the dark at 4°C. Seedling were germinated and grown in Petri dishes containing 1% of Phytoblend (Caisson Laboratories) and 0.5× Murashige & Skoog basal salts (Caisson Laboratories) at 22°C with long-day cycles (16-h light/8-h dark). The ecotype Columbia (Col-0, Col) was used as the wild type (WT) for transgenic studies. Mutants used in this study, *basl-2* (WiscDsLox264F02) and *basl-3* (SAIL_547_F11), were obtained from the Arabidopsis Biological Resources Center at the Ohio State University. *basl-3,* but not *basl-2,* is sensitive to Hygromycin selection, so was used as a genetic background for pMDC43 and R4pGWB510 complementation assay.

### Plasmid construction and plant transformation

The Gateway cloning technology (Invitrogen) was used for most DNA manipulations. The coding regions of BASL and specific subdomains were amplified by Phusion high-fidelity enzyme (New England BioLabs) with appropriate primers (published in Dong *et al.* (2009) and listed in Supplementary Table S1 at JXB online) and sub-cloned into the pENTR/D-TOPO vector (Invitrogen). To introduce point mutations, pENTR/D-BASL was used as a template and specific mutations were introduced by a QuickChange II XL site-Directed Mutagenesis Kit (Agilent Technologies). The binary vector pMDC43 (Curtis and Grossniklaus, 2003), in which the *35S* promoter was replaced with the *BASL* promoter, was used for expressing GFP-tagged full-length BASL and BASL_N, _I, _I2, _C, _NI, and _IC domains. To generate myristoylated GFP-BASL, the GFP fragment in the pMDC43 (containing the *BASL* promoter driving GFP-BASL) was replaced with Myr-GFP. The other orientation of Myr-BASL-GFP was generated by initial cloning into pENTR/D-*BASLpro*::Myr-BASL and subdomains, followed by recombination into pMDC107 (Curtis and Grossniklaus, 2003). To express BASL_NLSd, _NESd, and _NLSd/NESd in plants, R4pGWB510 (Nakagawa *et al.*, 2008) was used to recombine with pENTR/D-YFP-BASL variants and pDONR P4-P1r-*BASLpro* (Zhang *et al.*, 2015). All BASL-related constructs were driven by the *BASL* promoter for consistent experimenting. Plant transformations were carried out with the *Agrobacterium* (strain GV3101)-mediated floral dipping method (Zhang *et al.*, 2006). Subsequent selection of transgenic plants followed standard protocols.

### RT-PCR

Total RNAs were extracted from 7-days-post-germination (dpg) seedlings using an RNeasy Plant Mini Kit (Qiagen). The first-strand cDNAs were generated from 3 µg of total RNAs in a total volume of 20 µL reaction using the SuperScript^TM^ First-Strand Synthesis System (Invitrogen). PCR reactions were carried out using 0.5 µL of the reverse transcription resultant for a total volume of 20 µL. Gene-specific primers were used to evaluate the endogenous expression level of *BASL* and *18s rRNA* (primers listed in Supplementary Table S1 at *JXB* online) for 35-cycle or 25-cycle PCR reactions, respectively. 10 µL of PCR product from each were loaded for DNA electrophoresis.

### Confocal microscopy and image processing

In general, 2–3 dpg *Arabidopsis* cotyledons were used for confocal imaging on a Leica TCS SP5 II microscope. To outline the epidermal cells, seedlings were incubated in 10 µg/mL propidium iodide (PI, Invitrogen) for 10min. To mark the nucleus, seedlings were treated with 1 µg/mL DAPI (Invitrogen) in 1% Tween-20/phosphate-buffered saline staining solution for 10min. The excitation (Ex) and emission (Em) spectra for fluorescent proteins and PI staining were specified as: YFP, 514nm (Ex) and 520–540nm (Em); GFP, 488nm (Ex) and 501–528nm (Em); PI, 543nm (Ex) and 591–636nm (Em); DAPI, 360nm (Ex) and 456–460nm (Em). All image processing and quantification were performed or facilitated with Fiji software (http://fiji.sc/Fiji). Figure panels were assembled in Adobe Illustrator CS6.

### Quantification of stomatal development and patterning

The quantification protocol followed the previous publications (Dong *et al.*, 2009; Zhang *et al.*, 2015) with minor modifications. In general, more than 12 independent T2 transgenic lines were maintained for each transgene. For each transgenic event, two representative lines with stable YFP/GFP expression, and single insertions preferred, were established and pooled together for quantification analysis. High ‘stomatal index’ (number of stomata/total number of epidermal cells expressed as a percentage) describes stomatal over-production, and ‘clustered stomata’ (more than two stomata in direct contact) indicates the patterning defects [overriding the ‘one cell-spacing rule’ (Geisler *et al.*, 2003)]. Complementation of the *basl* phenotype was scored in dissected cotyledons of 5-dpg seedlings stained with PI. Similar central regions in the adaxial cotyledons were examined and imaged with an EC Plan-Neofluar (20×/0.5) lens on a Carl Zeiss Axio Scope A1 fluorescence microscope equipped with a ProgRes MF CCD camera (Jenoptik). Typically, about 1500–2000 epidermal cells were counted for phenotyping. Stomatal cells were scored as one of the four categories: pavement cells, stomata, clustered stomata, and small dividing cells as in Dong *et al.* (2009). Two key phenotypes of *basl* mutants (high stomatal index and clustered stomata) were used to evaluate the efficacy of transgenes in complementation assays. The numbers were subjected to normal probability tests and the OriginLab analysis, followed by the Mann–Whitney test (data point < 30).

### Quantification of BASL variant polarization

Quantification of protein polarity degree was described in Zhang *et al.* (2015) with slight modifications: 3-dpg seedlings were counterstained with PI before mounting and examined on the confocal microscope. A total of 30 GFP-positive cells from at least five cotyledons were selected for polarity quantification. Along the PI-stained cell periphery of the stomatal ACD cells, GFP intensity was measured from two equal-sized regions (covering about 25% of the perimeter) at the PM that expressed high or low GFP, respectively. Uneven protein distribution was measured by the ratio of the high GFP intensity value over the low intensity value. Typically, measurements from 30 cells were subjected to the Student’s *t* test (data point ≥30) using OriginLab software.

## Results

### BASL nuclear localization requires the nuclear localization signals, but export is nuclear export signal independent

**Fig. 1.**
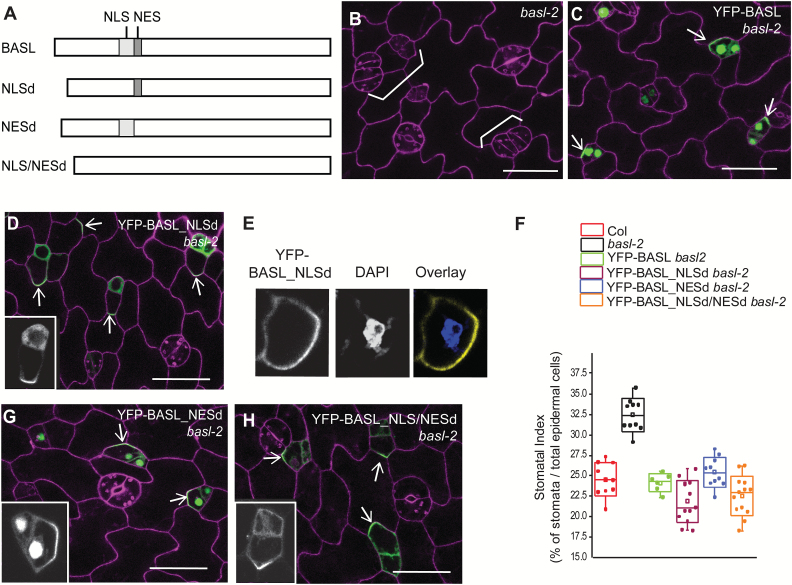
BASL requires the NLS for its nuclear import. (**A**) Diagram representing the 262 amino-acid WT BASL protein and sites of modifications. Light grey box indicates the putative NLS and the dark grey box marks the putative NES. NLSd, the NLS deleted in BASL; NESd, the NES deleted; NLS/NESd, the NLS and NES both deleted. (**B–D**) Confocal images of 2-dpg adaxial cotyledon to show that without the NLS, BASL is no longer restricted to the nucleus. Protein localization is shown in green and cells were outlined with PI staining (magenta). White brackets label clustered stomata or lineage cells. The clustered stomata in the loss-of-function mutant *basl*-2 (B) were complemented by expressing both YFP-BASL (C) and YFP-BASL_NLSd (D). White arrows point to protein polar accumulation. The inset in D shows the YFP channel only for a closer view of BASL_NLSd localization. Scale bars = 25 µm. (**E**) Confocal images of YFP-BASL_NLSd (yellow) in the cell counterstained with DAPI (blue), a fluorescent dye that binds to nucleic acids to show the nucleus, suggesting that BASL_NLSd is not expressed in the nucleus. (**F**) Phenotypic quantification of transgenic plants expressing YFP-tagged proteins. Stomatal index was calculated as the total number of stomata over the total number of epidermal cells and expressed as a percentage. The box plots were created by OriginLab. Each dot represents the stomatal index of an individual seedling (n = 10–12 seedlings, 5 dpg); for each seedling, about 150–200 cells (1500–2000 in total) were scored. This applies to other figures in which this format is used. Note the similar rescue effect of YFP-BASL_NLSd, _NESd, and _NLS/NESd in *basl-2* compared to that of YFP-BASL. All BASL-related constructs were driven by the *BASL* promoter. (**G–H**) Confocal images of YFP-BASL_NESd (G) and YFP-BASL_NLS/NESd (H) (green) in *basl-2* to show protein localization (green) and complementation (cells outlined with PI staining, magenta). BASL does not rely on the NES for nuclear export, but requires the NLS for nuclear import. Arrows indicate proteins polarized to cortical crescents. The insets show the YFP channel only. Scale bars = 25 µm.

Shortly after mitosis in early stomatal lineage cells, GFP-BASL expression is visible in the nucleus, and over time accumulates at the PM (Dong *et al.*, 2009). We previously showed that a mutant form (phospho-deficient) of BASL protein unable to be exported from the nucleus fails to rescue the *basl* mutant (Zhang *et al.*, 2015); therefore, we looked for BASL motifs that might mediate its partitioning between the nucleus and at the PM. In principle, cytoplasmically synthesized proteins are imported into the nucleus via the nuclear localization signals (NLSs) of the protein. We conducted NLS prediction using the cNLS mapper (Brameier *et al.*, 2007) (http://nls-mapper.iab.keio.ac.jp/cgi-bin/NLS_Mapper_form.cgi) and found that BASL contains two putative monopartite NLSs (positioned at amino acids 51–60 and 61–68, shown in red in Supplementary Fig. S1A at *JXB* online). We constructed YFP-BASL_NLSd (Fig. 1A, missing both NLSs, amino acids 51–64 deleted) for a localization and complementation assay. In contrast to YFP-BASL, which recapitulated the localization pattern of the previously published GFP-BASL (Dong *et al.*, 2009) and complemented the loss-of-function *basl-2* mutant (Fig. 1B, C), YFP-BASL_NLSd did not show obvious nuclear enrichment, but instead was diffuse in the cytoplasm and polarized at the cell cortex (Fig. 1D). DAPI nucleic acid staining confirmed that YFP-BASL_NLSd was not retained in the nucleus (Fig. 1E). Interestingly, YFP-BASL_NLSd was sufficient to rescue *basl*-2 at the scale of our phenotypic measurements, including stomatal production (stomatal index) and patterning (clustered stomata) (Fig. 1B versus Fig. 1D, quantification in Fig. 1F and see Supplementary Fig. S1B, C at *JXB* online). These data are consistent with our previous finding that BASL functioned primarily at the cortical polarity site (Dong *et al.*, 2009).

Nucleocytoplasmic shuttling often requires a leucine-rich nuclear export signal (NES), which is recognized by the importin family to cross the nuclear envelope (la Cour *et al.*, 2004). The NetNES predictor (http://www.cbs.dtu.dk/services/NetNES-1.1/) suggested the possible existence of a weak NES in BASL (see Supplementary Fig. S1 at *JXB* online, amino acid position 65–71, scored at 0.4–0.5). We deleted this region to generate YFP-BASL_NESd. Confocal examination showed that, even without the putative NES, BASL protein still maintained its typical localization and function in plants (Fig. 1F, G). Simultaneous deletion of the NLSs and NES, YFP-BASL_NLS/NESd, produced a protein mostly mimicking YFP-BASL_NLSd in localization and function (Fig. 1F, H). Together, our results indicate that the NLSs do promote nuclear localization, but export of BASL does not rely on its putative NES. Further, nuclear retention of BASL is not absolutely required for its peripheral polarity or for its function in stomatal ACDs.

### BASL cortical polarization requires conserved motifs within the C-terminal domain

**Fig. 2.**
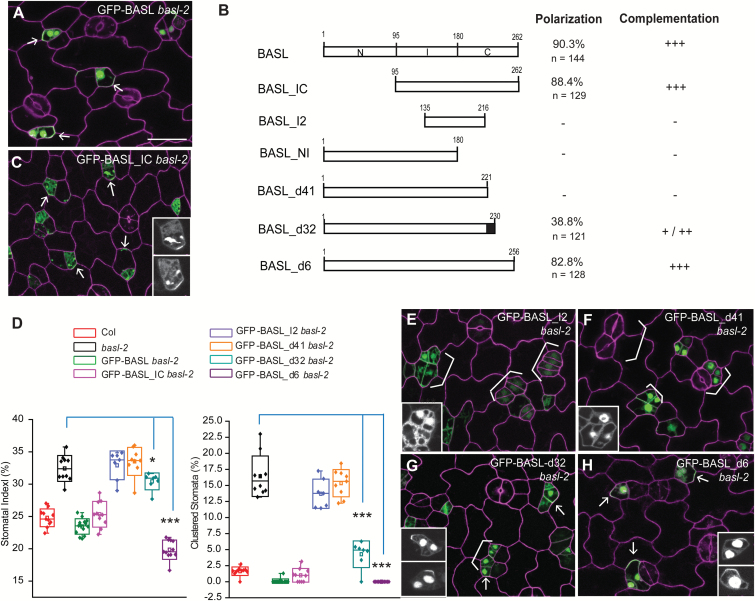
A small motif is required for BASL polarization. (**A**) Confocal image to show GFP-BASL localization (green) in a 2-dpg adaxial *basl-2* cotyledon counterstained with PI (magenta). White arrows mark evident protein polarization. Scale bars = 25 µm. The other panels are at the same scale. (**B**) Bar diagrams describe BASL truncations tested for subcellular localization and function in plants. The ‘polarization’ column displays the percentage of cells showing visible polarity in GFP-positive cells (n). Their effects in functional complementation are summarized as no effects (-), weak (+), medium (++), and strong (+++). The black box highlights the critical motif that recovered BASL polarity. (**C**) Confocal image of GFP-BASL_IC (green) in a 2-dpg *basl-2* cotyledon counterstained with PI (magenta). Note the polar accumulation of BASL_IC (white arrows) in stomatal ACDs and its capability of complementation. The insets show the GFP channel only. (**D**) Box plots show quantification of stomatal phenotype in different genotypes. About 1500–2000 total cells were collected from 5-dpg cotyledons (n = 10–12 from two independent transgenic lines). BASL_I2 and BASL_d41 failed to rescue *basl*-2; BASL_d32 partially rescued *basl*-2 and BASL_d6 efficiently complemented *basl-2.* (Mann–Whitney test, *P < 0.05, ***P < 0.001). (**E–H**) Confocal images to show protein localization (green) of GFP-BASL_I2 (E), GFP-BASL_d41 (F), GFP-BASL_d32 (G), and GFP-BASL_d6 (H) in *basl-2*. The 2-dpg seedlings were stained with PI (magenta). White brackets indicate clustered stomata lineage cells, indicating failure to complement. Arrows show protein polarization in G and H. The small motif between BASL_41 and BASL_d31 (black box in B) contains information for BASL polarization and function. The insets show the GFP channel only for detailed protein localization.

Perhaps the most striking property of BASL is its polarized cortical localization in asymmetrically dividing stomatal cells (Fig. 2A, 90.3% GFP-positive cells show protein polar accumulation, n = 144 in 2-dpg seedlings). We previously demonstrated that the C-terminal two-thirds of BASL (amino acids 95–262, BASL_IC) was sufficient for cortical localization, for polarization, and for function (Fig. 2B–D, 88.4% cells show GFP polar accumulation, n = 129), but that regions I or C alone were not (Dong *et al.*, 2009). We further dissected BASL_IC, creating a fragment BASL_I2 (amino acids 135–216) that overlapped the junction between I and C (Fig. 2B). This fragment was not sufficient to polarize or rescue *basl-2* (Fig. 2D, E). These data suggested that BASL polarization requires information from the regions beyond BASL_I but within BASL_IC, including amino acids 95–135 and/or 216–262 (Fig. 2B). Because our previous data showed BASL_NI (amino acids 1–180, containing 95–135) was not functional or polarized (see Supplementary Fig. S2A, B at *JXB* online), we focused our analysis on the carboxyl terminal region (amino acids 221–262, Fig. 2B).

**Fig. 3.**
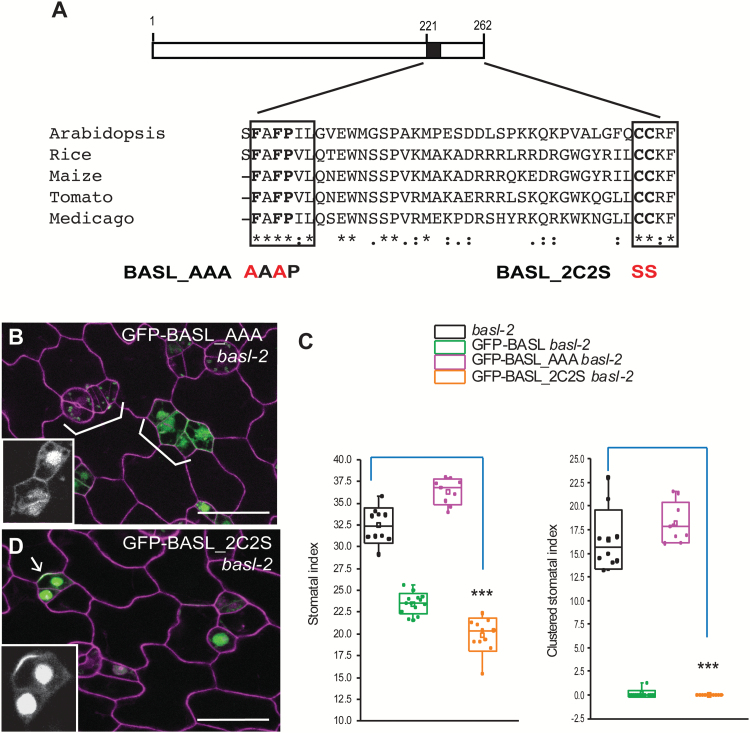
Two conserved amino acids are critical. (**A**) Boxed diagram demonstrates the conserved C-terminal domain of BASL. The black box marks the critical small motif for BASL polarity formation. The last 41 amino acids align with a few BASL-like proteins from other plant species. Two conserved motifs, FAFPI/VL and CCR/KF, are indicated with boxes. BASL_AAA and BASL_2C2S were generated by mutating FAF to AAA and CC to SS, respectively. Red indicates site mutations. (**B**) Confocal image to show that GFP-BASL_AAA (green) totally lost polarization. Cell outlines were stained with PI (magenta). White brackets point to clustered stomata, suggesting the *basl-2* phenotype was not rescued. Scale bar = 25 µm. (**C**) Quantification of stomatal phenotype in 5-dpg adaxial cotyledons. GFP-BASL_2C2S, but not GFP-BASL_AAA, complemented the stomatal defects in *basl-2.* (Mann–Whitney test, ***P < 0.001 compared to *basl*-2). (**D**) GFP-BASL_2C2S (green) is polarized and functional in *basl-2*. Insets in B and D show representative protein localization (GFP channel only). Arrow points to protein polarization. Scale bar = 25 µm.

Deletion of the C-terminal 41 amino acids (amino acids 222–262, GFP-BASL_d41) completely eliminated polarization and the protein accumulated instead in the nucleus and cytoplasm (Fig. 2F). Concomitantly, GFP-BASL_d41 was unable to complement *basl-2* (Fig. 2D), indicating that the last 41 amino acids are indispensable. To identify critical motifs within this essential domain, we blasted the last 41 amino acids of BASL against the phytozome database (http://phytozome.jgi.doe.gov/pz/portal.html). This search revealed blocks of conservation with other plant proteins (Fig. 3A), particularly around two motifs: FAFPI/VL (FxFP) and CCR/KF (boxed in Fig. 3A). The FxFP motif can mediate interactions between MAPKs and their substrates (Jacobs *et al.*, 1999) and CCR/KF motifs at the C-terminus can be palmitoylated to retain proteins at membranes (Tani and Hannun, 2007; Mattison *et al.*, 2012). These were provocative motifs given the membrane association of BASL and the importance of MAPK regulation in stomatal development (Wang *et al.*, 2007; Lampard *et al.*, 2008; Lampard *et al.*, 2009).

To test whether the FxFP motif (black box in Fig. 2B) was necessary or sufficient for BASL activity, we first extended BASL_d41 by nine amino acids so that the motif was maintained. GFP-BASL_d32 significantly recovered polarization (Fig. 2G, 38.8% cells showed GFP cortically polarized, n = 121) and complementation abilities (Fig. 2D). We then did the reciprocal experiments of eliminating only the FxFP site in an otherwise WT BASL (GFP-BASL_AAA, where the two phenylalanines were substituted by alanines FAF > AAA, Fig. 3A). This version was no longer polarized, nor was it able to rescue the *basl-2* phenotype (Fig. 3B, C).

The role of the CCR/KF motif is less clear. GFP-BASL_d6 (missing this motif in the last six amino acids) was similar to WT in its ability to polarize and complement the mutant (Fig. 2H, D, polarity shown in 82.8% cells, n = 128). GFP-BASL_2C2S, with the final two cysteines replaced by serines (Fig. 3A), maintained polarization activity and rescued *basl*-2 mutants, comparable to the WT (Fig. 3D, quantification in Fig. 3C). Therefore, regarding BASL’s localization at the PM, we have no evidence that direct palmitoylation of BASL drives membrane association.

### Tethering BASL and subdomains to the plasma membrane

Deletions and site mutations are useful to assess the contribution of specific motifs to BASL localization and function, but the subsequent mislocalization often attenuates our ability to assay polarity at the PM. Artificially tethering the BASL protein or domains would explicitly demonstrate how parts of BASL might function at the PM. In addition, many models for polarization require protein movement on and off the membrane (Altschuler *et al.*, 2008; Slaughter *et al.*, 2009). By taking advantage of the myristoylation (Myr) modification that permanently attaches the target protein to the PM (Martin *et al.*, 2011), we tested aspects of BASL polarization and function at the PM and its biological significance in the nucleus (summarized in Supplementary Table S2 at *JXB* online).

**Fig. 4.**
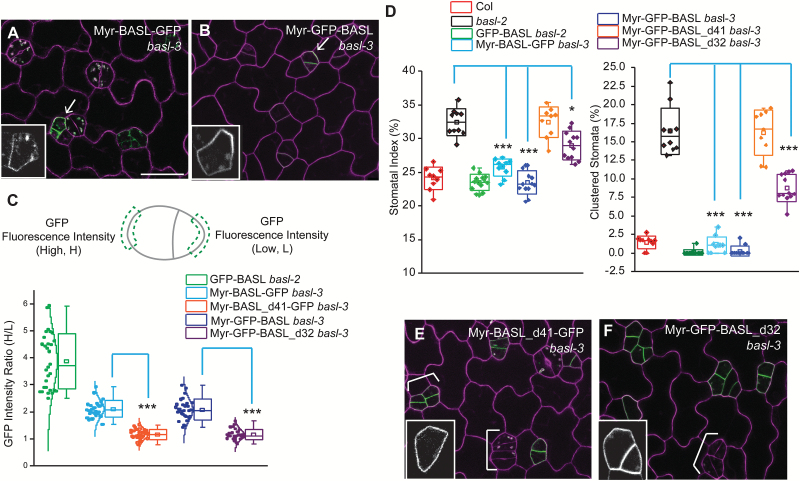
BASL’s function at the PM requires the small motif. (**A–B**) Confocal images of Myr-BASL-GFP (A) and Myr-GFP-BASL (B) (green) in *basl-3*. Both showed polar distribution at the PM and rescued the mutant. Magenta shows the PI-stained cell walls. Arrows point to polar accumulation of proteins. Insets display the GFP channel only. Scare bar = 25 µm. Other confocal images are at the same scale. (**C**) Quantification of protein polarization. Myr-BASL was found weakly polarized, but protein polarization was not detectable in Myr-BASL_d41 or Myr-BASL_d32. The diagram (top) demonstrates how protein polarity degree is calculated. GFP intensity was collected from two same-sized segments from a stomatal ACD pair. The ratio of H/L (H taken from the large cell and L taken from the small cell) reflects the protein polarity degree. The box plot (bottom) demonstrates differentially polarized proteins. The bell-shaped notches describe normally distributed individual values (n = 30 GFP-positive stomatal ACD pairs from each transgenic line). Note the loss of polarity in Myr-BASL_d41-GFP (Mann–Whitney test, ***P < 0.001 compared to Myr-BASL-GFP) and Myr-GFP-BASL_d32 (***P < 0.001 compared to Myr-GFP_BASL). (**D**) Box plots show quantitative analysis of stomata phenotype. Note the rescuing effect of Myr-BASL-GFP and Myr-GFP-BASL, and the partial rescuing by Myr-GFP-BASL_d32 (Student’s *t* test, *P < 0.001, ***P < 0.05, compared to *basl-2*), but not of Myr-GFP-BASL_d41, suggesting that without the black box (in Fig. 3A), BASL cannot function at the PM. (**E–F**) Confocal images showing the localization of Myr-BASL_d41-GFP (E) and Myr-GFP-BASL_d32 (F) in *basl-3*. Both are found at the PM, but not polarly. White brackets highlight cluster stomata lineage cells. Representative protein localization was shown in the insets. Note clustered stomatal lineage cells (white brackets) were not present in A and B, suggesting the failures of transgene complementation.

A Myr-targeted peptide sequence (Greenwood and Struhl, 1999) was fused to the N-termini of BASL and its variants. We found that the myristoylated full-length BASL did not show obvious nuclear expression and that both orientations, Myr-BASL-GFP and Myr-GFP-BASL, were somewhat polarly distributed along the PM (Fig. 4A, B, polarity quantification in Fig. 4C) and largely rescued *basl-3* mutants (complementation quantified in Fig. 4D). *basl-3* is indistinguishable from *basl-2* in stomatal phenotypes (see Supplementary Fig. S2C–E at *JXB* online), but the *basl-3* T-DNA confers an easier selection for the expression vectors used in this study, and therefore was equivalently used for complementation assays. These results suggest that the on-and-off membrane mechanism may not be necessary for BASL polarization and function. We then generated Myr-BASL_d41 and Myr-BASL_d32 and found both are localized to the PM, but no obvious polar distribution could be detected (Fig. 4E, F). Myr-BASL_d41, similar to BASL_d41, did not complement the stomatal defects in *basl-3*. Myr-GFP-BASL_d32 was also not discernably polarized (in contrast to GFP-BASL_d32, Fig. 2G), but, interestingly, it partially rescued the *basl-3* mutant (Fig. 4D). It is possible that the higher overall levels of membrane-associated Myr-BASL_d32 obscured the polarization of this construct, but the functional motif in BASL_d32, that is FxFP, was sufficient to contribute at least partially to protein activity.

**Fig. 5.**
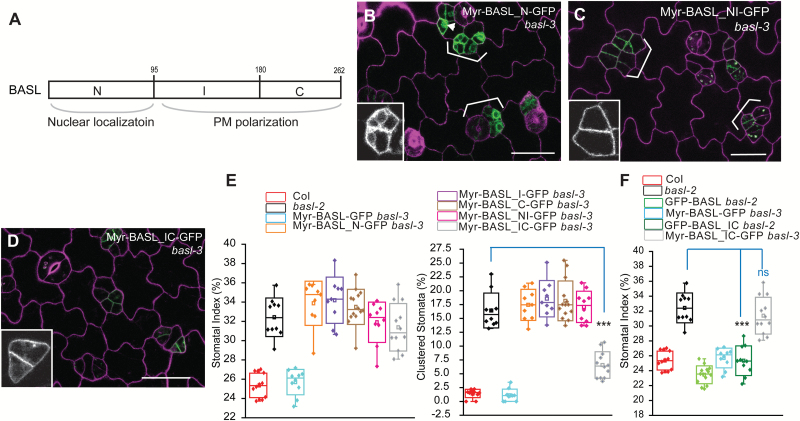
Localization and function of myristoylated BASL domains. (**A**) Box diagram shows BASL subdomains, N (N-terminal), I (Internal), and C (C-terminal). BASL_N was found to direct nuclear localization and BASL_IC mediates polarization at the PM. (**B–D**)Confocal images show localization of fusion proteins in *basl*-3 (green), Myr-BASL_N-GFP (B), Myr-BASL_NI-GFP (C), and Myr-BASL_IC-GFP (D). Cells were outlined with PI staining (magenta). Representative protein localization is shown with the GFP channel only (insets). Brackets highlight stomatal clustering. Note the Myr-BASL_N-GFP protein in the cytoplasm and some in the nucleus (inset in B), likely due to the antagonistic localization mechanisms to the nucleus and to the cortical membrane. Scale bar = 25 µm. (**E**) Box plots show quantification of stomatal phenotypes in complementation. Myr-BASL_IC partially rescued *basl*, in particular in terms of clustered stomata (Student’s *t* test, ***P < 0.0001 compared to *basl-2*). (**F**) Box plots show the comparison of transgene complementation. GFP-BASL_IC, but not Myr-BASL_IC-GFP, most effectively rescued the *basl* null mutant. Mann–Whitney test, *** *P <* 0.001 compared to *basl-2* and ‘ns’ indicates ‘not significant’.

**Fig. 6.**
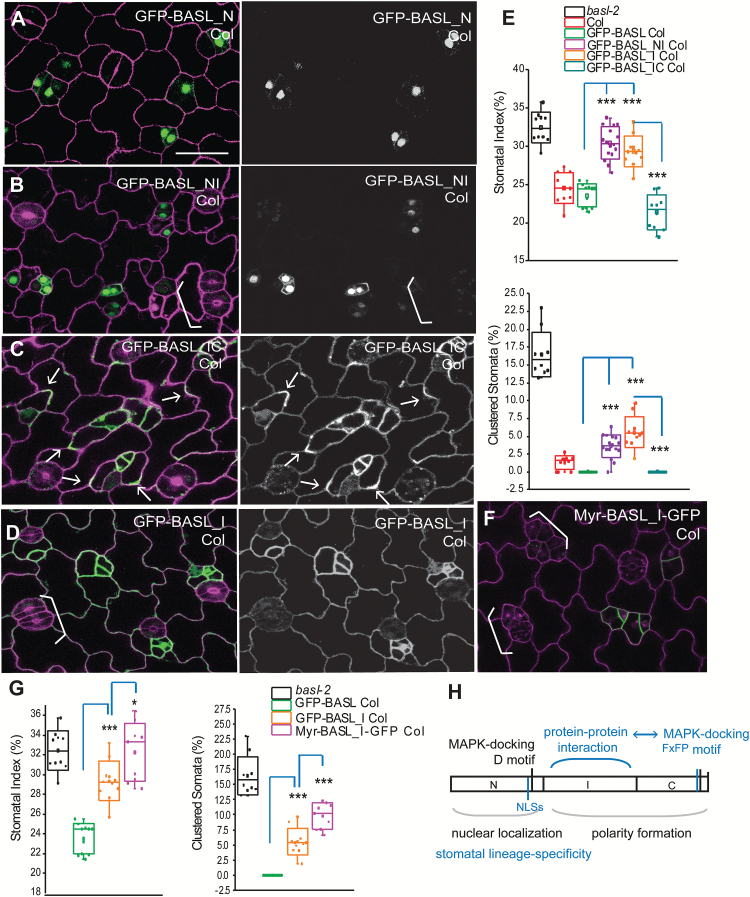
Overexpression of BASL subdomains driven by the endogenous promoter. (**A**) Confocal image of 2-dpg adaxial cotyledon overexpressing GFP-BASL_N (green) in the WT (Col). Left, protein localization shown in green and cell shape marked by PI staining (magenta). Right, the GFP channel only shows the expression pattern of GFP-tagged proteins. The other panels (B–D) are similarly formatted and at the same scale. Scale bar = 25 µm. (**B**) Overexpression of GFP-BASL_NI. White bracket indicates clustered stomata, suggesting GFP-BASL_NI is interfering with the endogenous BASL function. (**C**) Overexpression of GFP-BASL_IC. Arrows indicate polar accumulation of the protein. Note the broader expression in pavement cells (right), suggesting that BASL_IC is not degraded in pavement cells and the WT BASL protein is likely subject to protein degradation there. (**D**) Overexpression of GFP-BASL_I. Note clustered stomata (white bracket) and GFP expression in pavement cells. Similar to BASL_NI, BASL_I also generated dominant negative effects. (**E**) Box plots showing quantification of stomatal phenotypes in overexpression lines. Note the dominant negative effect of GFP-BASL_NI and GFP-BASL_I (relative to GFP-BASL) and the dominant negative effect of GFP-BASL_I released in GFP-BASL_IC plants. Mann–Whitney test, ***P < 0.001. (**F**) Overexpression of Myr-BASL_I-GFP. The dominant negative effect was evident (white brackets highlighting clustered stomata), indicating that the PM pool of BASL_I is interfering, possibly by competing with the endogenous protein to bind to partner/s at the PM. (**G**) Box plots comparing the dominant negative effect of GFP-BASL_I and Myr-BASL_I-GFP (relative to GFP-BASL). Mann–Whitney test, *** *P* < 0.001; * *P* < 0.01). (**H**) Diagram to summarize the proposed functions of BASL subdomains. Blue colour indicates the new findings from this study. The minimal polarity complex at the PM may require the combined functions of a novel (currently unidentified) BASL_I-mediated protein–protein interaction and BASL_C-mediated interaction with the YDA MAPK module. BASL-N, through the NLSs, directs the nuclear localization and also contributes to stomatal lineage-specific expression of the BASL protein.

We continued to utilize Myr as a tool to test BASL subdomains (Fig. 5A). The N-terminal domain (BASL_N) directs nuclear localization and cannot rescue *basl-2* null mutants (Dong *et al.*, 2009). When Myr-BASL_N was fused to GFP, it was found to be localized primarily in the cytoplasm (Fig. 5B) and a small number of cells even showed nuclear expression (arrowhead in Fig. 5B). Such localization might result from the antagonistic activity between the NLSs in BASL_N and the Myr modification. However, Myr-BASL_NI (with the addition of the I domain) mainly localized to the PM (Fig. 5C), indicating that BASL_I might promote the PM association. Previously we showed that BASL interacts with the MAPKKK YDA via three MAPK-docking domains (Zhang *et al.*, 2015), but none of which resides in BASL_I (Fig. 6H). Therefore, we propose that BASL_I might interact with new partner/s to associate BASL with the PM.

Without the N domain, BASL_IC was mainly cytoplasmic but polarized efficiently at the cell cortex and it fully rescued *basl* mutants (see Supplementary Fig. S2B at *JXB* online) (Dong *et al.*, 2009). But, once myristoylated, association of the BASL_IC with the PM lacked obvious polarization (Fig. 5D) and its ability to rescue the *basl* mutant was significantly impaired (Fig. 5E). The decreased complementation capability of Myr-BASL_IC relative to that of BASL_IC (Fig. 5F) might be explained by the greatly reduced polarization and/or the decreased partition in the cytoplasm, where the activating MPK3/6 are enriched (Zhang *et al.*, 2015).

We also assayed other subdomains, namely, the cytoplasmic BASL_I and BASL_C (Dong *et al.*, 2009). Our data showed that these Myr-versions were predominantly enriched at the PM (see Supplementary Fig. S2F, G at *JXB* online) and were not effective in complementing the *basl* phenotype (Fig. 5E).

### Using tethered BASL subdomains to assay for putative interaction platforms

At the cortical polarity site, BASL scaffolds YDA and MPK3/6 and there is mutual positive reinforcement of polarity through interactions among the constituent members (Zhang *et al.*, 2015). Loss of *YDA* did not fully abolish BASL polarity (Zhang *et al.*, 2015), however, suggesting that other partners are required to polarize BASL. While identification of such partners is beyond the scope of this paper, hints of their target sites on BASL can be revealed by misexpressing small domains and assaying for dominant negative effects. The rationale for this approach is that the overexpressed target site will outcompete endogenous BASL for interaction with the partner, leading to failure of BASL to polarize or function in generating asymmetry (for examples, see Roth *et al.*, 1998; Zhou *et al.*, 2015).

We introduced *BASL* promoter-driven GFP-tagged BASL subdomains, including N, I, I2, C, NI, and IC (Fig. 6 and see Supplementary Fig. S3 at *JXB* online), into the WT Col plants. We found that they showed a similar localization pattern as they did in *basl* mutants. The subdomains containing the N fragment, that is, BASL_N and BASL_NI, showed obvious nuclear enrichment (Fig. 6A, B). BASL-IC was cytoplasmic and polarized at the cortex (Fig. 6C) and BASL_I, _I2, and C were primarily localized in the cytoplasm (Fig. 6D, and see Supplementary Fig. S3A, B at *JXB* online).

We then examined stomatal formation and patterning in transgenic plants. Elevated expression of the full-length *BASLpro::*GFP-BASL (an extra copy in WT) did not generate obvious alterations (see Supplementary Fig. S3C, D at *JXB* online). Overexpression of the subdomains BASL_NI and BASL_I, however, induced overproduction and clustered stomatal lineage cells, resembling that of the *basl* mutants (Fig. 6B, D versus Fig. 1B; quantifications in Fig. 6E). To exclude possible transgenic silencing, we conducted reverse transcription (RT)-PCR to show that endogenous *BASL* was normally expressed (see Supplementary Fig. S4 at *JXB* online). These results suggested that BASL_I generated a dominant negative effect likely competing with the endogenous BASL to bind to functional partner/s. Because the interaction between BASL and YDA or MPK3/6 uses motifs outside of this region (Zhang *et al.*, 2015) and we did not find a physical interaction between BASL_I and YDA or MPK3/6 in a yeast-two-hybrid assay (data not shown), we suspect that BASL_I may interact with a novel partner/s for its function.

Interestingly, combining the C-domain with BASL_I eliminated its dominant negative effect, producing stomata comparable to those of the WT (BASL_IC versus BASL_I, Fig. 6E). These data indicate that the BASL_C domain suppresses BASL_I’s interference, likely by bringing in interacting partners to form a functional complex, which is manifested by polarity recovery of BASL_IC (Fig. 6C). Overexpressing other subdomains (BASL_N, BASL_I2, and BASL_C) did not produce obvious phenotypes in stomatal development (see Supplementary Fig. S3D at *JXB* online).

Because GFP-BASL_I was found in the cytoplasm and at the PM (Fig. 6D), to further dissect where BASL_I confers dominant negative effects we overexpressed Mry-BASL_I-GFP. Intriguingly, Mry-BASL_I produced a stronger dominant negative effect than BASL_I did (Fig. 6F, G), suggesting that it is at the PM that critical BASL_I /partner interactions take place and outcompete endogenous BASL (Fig. 6H). Such an effect is likely due to the tight PM association rather than the protein levels of Myr-BASL_I, given that BASL_I is generally expressed more abundantly in plants (Fig. 6D versus Fig. 6F). The dominant negative phenotype is specific to the BASL_I region in that neither Myr-BASL_N or Myr-BASL_C created dominant negative effects (Supplementary Fig. S3F, G, quantified in Supplementary Fig. S3D at *JXB* online).

### The BASL N-terminus regulates protein accumulation

It was previously noted that the *BASL* promoter activity had a broader expression pattern than that of the protein fusion (Dong *et al.*, 2009). The BASL protein was primarily found in the stomatal linage cells (see Supplementary Fig. S3C at *JXB* online), but the BASL promoter had low, but clear, expression in the epidermal pavement cells as well (see Supplementary Fig. S3E at *JXB* online). Interestingly, the overall expression pattern of BASL subdomains suggests that the N domain of BASL confers stomatal lineage-specific expression because neither BASL_N nor BASL_NI were found outside of the stomatal lineage (Fig. 6A, B). However, without the N domain, the subdomains of BASL_I, I2, C, or IC showed a much broader pattern—they were also expressed in the pavement cells (Fig. 6C, D; see Supplementary Fig. S3A, B at *JXB* online)—which was similar to that of the *BASL* promoter (see Supplementary Fig. S3E at *JXB* online). These data suggest that the down-regulation or degradation of BASL protein in pavement cells might be mediated by the BASL_N domain.

## Discussion

### Nuclear localization and potential degradation mechanisms for cell type-specific expression of BASL

The expression of BASL was initially seen in the nuclei of the protodermal cells in *Arabidopsis* seedlings (as early as 16 hours after germination), before cortical polarization was evident (Dong *et al.*, 2009). Our previous studies suggested that BASL transiting through the nucleus might serve as a mechanism by which it could be phosphorylated and activated by MPK3/6 in the nucleus (Zhang *et al.*, 2015). Here our data showed that, without the NLSs, BASL remains in the cytoplasm, but it is still able to be polarized and to function. We hypothesize that BASL can be phosphorylated by cytoplasmic MAPKs or other kinases in the cell [localization of MAPKs in plant cells reported in Samajova *et al.* (2014)]. Proteins smaller than 60 kD can diffuse out of the nucleus freely (Laba *et al.*, 2014), which explains why BASL (below 30 kD) does not rely on the predicted NES for nuclear export. It is also possible that an unknown interacting protein transports BASL out of the nucleus in an NES-independent manner.

Although BASL’s function within the nucleus is still mysterious, that BASL_N restricts BASL expression to the stomatal lineage cells (Fig. 6 and see Supplementary Fig. S3 at *JXB* online) but BASL_IC is also found in pavement cells suggest that this compartment (nucleus) might be involved in a protein degradation process in pavement cells. Alternatively, motifs in BASL_N outside of the NLS could be targeted directly by the protein degradation system. Protein degradation is regulated by a highly conserved pathway, the ubiquitin–proteasome pathway (Callis and Vierstra, 2000). Preliminary analysis of the BASL protein sequence indeed suggested the possible existence of the ubiquitination sites with high confidence scores (data not shown). Future studies are required to elucidate whether ubiquitination and the proteasome degradation system mediate BASL protein abundance in the stomatal lineage cells, and which sites in BASL are ubiquitinated.

### BASL_I and possible function at the cortical polarity site

Overexpression of Myr-BASL_I at the PM created a strong dominant negative effect in stomatal development, suggesting critical protein–protein interaction mediated by BASL_I at the PM. The dominant negative effect of BASL_I being released by the integration of BASL_C also indicates that BASL_I-mediated interacting protein/s contributes partially to cell polarity and asymmetric division, and that the minimal functional complex may require both BASL_I- and BASL-C-mediated interacting proteins (Fig. 6H). The discovery of the FxFP MAPK DEF-docking motif in BASL_C led to the recognition of two other MAPK D-docking motifs in BASL (Fig. 6H) and a link to MPK3/6, which can phosphorylate BASL and allow for its polarization at the cell cortex (Zhang *et al.*, 2015). These MAPK docking motifs also seemed to mediate the interaction with the MAPKKK YDA, such that both YDA and MPK3/6 participate in the BASL polarity complex in the stomatal ACD cells (Zhang *et al.*, 2015). However, what binds to BASL_I remains unknown. Thus, our studies urge the discovery of interacting partners of BASL_I at the PM and further investigation of how the BASL-YDA-MPK3/6 module may cross talk with BASL_I-interacting proteins to function together in stomatal ACD.

In summary, in this study we characterized subdomains of the BASL polarity protein in detail. Although BASL protein is not large in size (262 amino acids), our domain analyses revealed that it is subjected to multiple interesting regulatory mechanisms for its localization and function (Fig. 6H). Our results support PM localization of BASL as being critical for its function in stomatal lineage asymmetric cell division. These findings inspire us to search for new partners and to investigate multiple pathways that are associated with specific BASL subdomains in cell polarity and ACD in plants.

## Supplementary material

Supplementary data are available at *JXB* online.

**Figure S1.** The predicted NLS and NES in the BASL protein.

**Figure S2.** Localization and function of BASL subdomains.

**Figure S3.** Overexpression of BASL subdomains by the *BASL* promoter.

**Figure S4.** Semi-quantitative RT-PCR for the endogenous expression of *BASL* in the overexpression lines of BASL subdomains.

**Table S1.** Primers used in this study.

Supplementary Data
