# Supplementary material for: Fine-scale dissection of the subdomains of polarity protein BASL in stomatal asymmetric cell division
Source: J Exp Bot. 2016 Jul 15;67(17):5093–103. doi: 10.1093/jxb/erw274 (PMC5014157; doi:10.1093/jxb/erw274)
Supplement: Supplementary Data [file supp_67_17_5093__index.html]

Fine-scale dissection of the subdomains of polarity protein BASL in stomatal asymmetric cell division — Fine-scale dissection of the subdomains of polarity protein BASL in stomatal asymmetric cell division — Supplementary Data 

# Fine-scale dissection of the subdomains of polarity protein BASL in stomatal asymmetric cell division

## Supplementary Data

Data files

- supplementary\_figures\_S1\_S4\_Tables\_S1\_S2.pdf - Supplementary Data
