# Supplementary material for: Fine-scale dissection of the subdomains of polarity protein BASL in stomatal asymmetric cell division
Source: J Exp Bot. 2016 Jul 15;67(17):5093–103. doi: 10.1093/jxb/erw274 (PMC5014157; doi:10.1093/jxb/erw274)
Supplement: Supplementary Data [file supp_erw274_supplementary_figures_S1_S4_Tables_S1_S2.pdf]

Fine-scale dissection of the subdomains of polarity protein BASL in stomatal asymmetric cell division

SUPPLEMENTARY FIGURES AND FIGURE LEGENDS

Figure S1.

A

```
>BASL
  1 MASQWTIPKL VTWRVRDWAS CFLACKIPLD GDEDGANNNG NTTNNNNLTF
 51 KRIKRKIKST KKKRSERKLS LSPPGTRHHH LHLRSSSVSP TTSGSQHRRL
101 SWPQPPVSEE SGFIVFCFDR EDGGFDVVKE GKQEKKETES SSEKSPRTVN
151 RKLIYGDQGV GGTEKNNSPE TKGTEQDQND NTSCQGTKDV SSDVTERTKE
201 EEDIDASDKS SGSSHSDEGR GSFAFPILGV EWMGSPAKMP ESDDLSPKKQ
251 KPVALGFQCC RF
```

B **C**

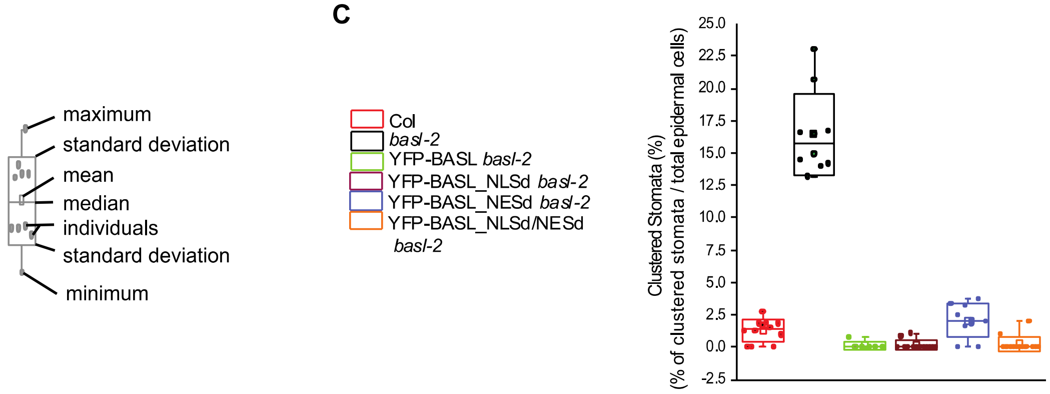

Figure S1. The predicted NLS and NES in the BASL protein.

(A) Two predicted monopartite NLSs are in red (51-60 and 61-68). Italic indicates the predicted NES (65-71). Green box labels the NLSs being deleted to generate BASL_NLSd and blue box marks the region deleted to generate BASL_NESd.

(B) Explanation of values in boxed plots.

(C) Phenotype quantification of clustered stomatal index in designated genotypes. The values were calculated by the percentage of the total number of stomata in direct contact over the total number of epidermal cells.

Figure S2.

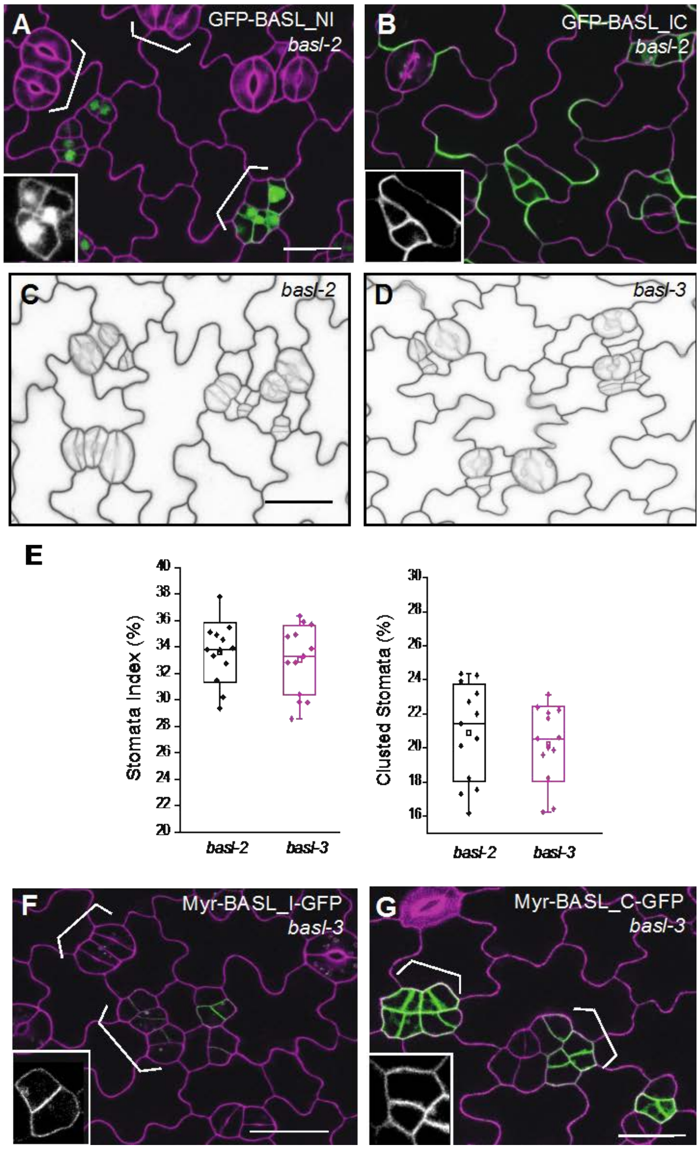

Figure S2. Localization and function of BASL subdomains.

(A-B) Confocal image to show 2-dpg adaxial cotyledons expressing fusion proteins in loss of *BASL* plants: GFP-BASL_NI *basl-2* (A), GFP-BASL_IC *basl-2* (B)

(C-E) Stomatal phenotype and quantification of *basl-2* (C) and *basl-3* (D). Confocal images were taken from 2-dpg seedlings and quantification was performed on 5-dpg cotyledons (E).

(F-G) Myr-BASL_I-GFP *basl-3* (F), and Myr-BASL_C-GFP *basl-3* (G). Protein localization is shown in green and cell outlines are marked with PI stain (magenta). Brackets indicate clustered stomata, a feature of *basl* mutants. Scale bars = 25 µm.

Figure S3.

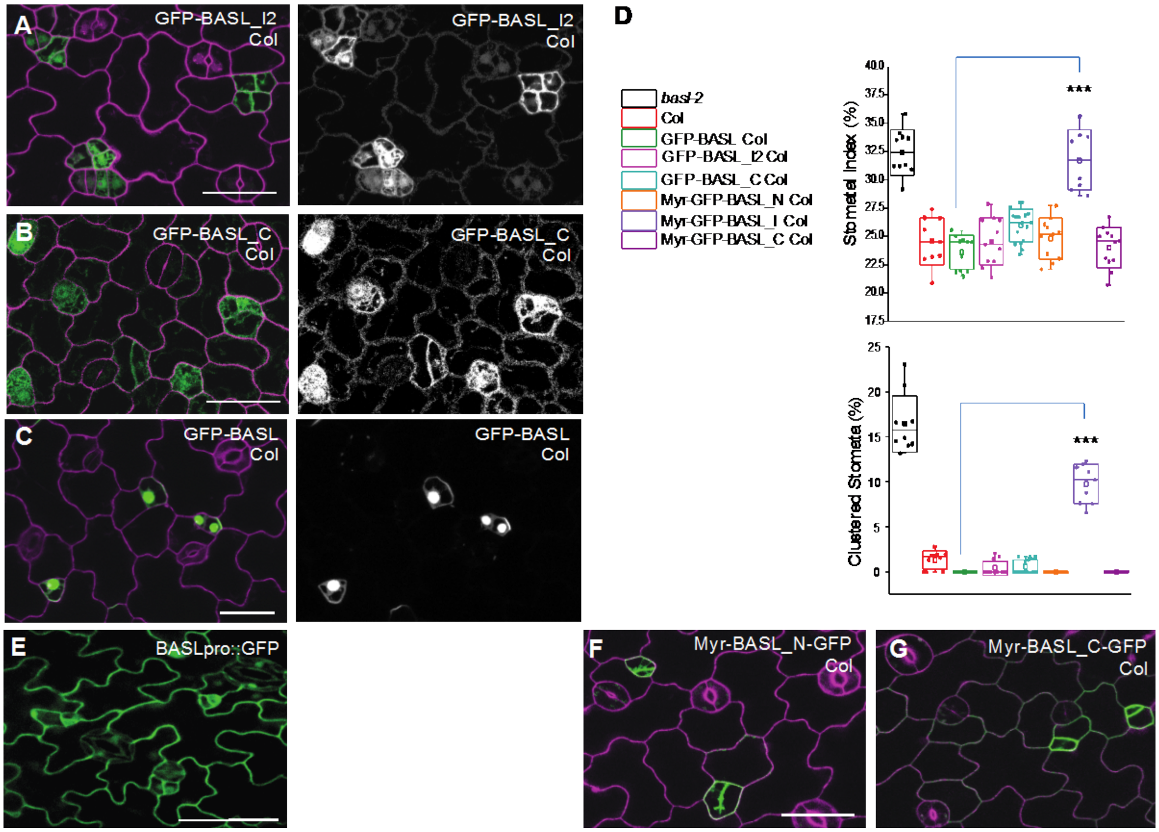

Figure S3. Overexpression of BASL subdomains by the *BASL* promoter.

(A-C) Confocal images of 2-dpg adaxial cotyledons overexpressing GFP-tagged BASL (A), BASL_I2 (B) and BASL_C (C). Left, seedlings were counterstained with PI to show GFP (green) and cell shape (magenta). Right, the GFP channel only to show stomatal development and patterning. Scale bars = 25 μm. Others are at the same scale.

(D) Quantification of stomatal phenotypes in overexpression plants. Two representative lines were picked up for each transgene and 10-12 seedlings on 5-dpg (1500-2000 cells in total) were used for quantification. The values in the box plots were explained in Fig. 1F. Note the dominant negative effect generated by Myr-GFP-BASL_I, but not by the others. Mann Whitney test, *** $P < 0.001$, relative to GFP-BASL.

(E) The transcriptional activity of *BASL* promoter is indicated by GFP expression (green).

(F-G) Confocal images of Myr-BASL_N-GFP (F) and Myr-BASL_C-GFP (G) in 2-dpg Col cotyledons.

Figure S4.

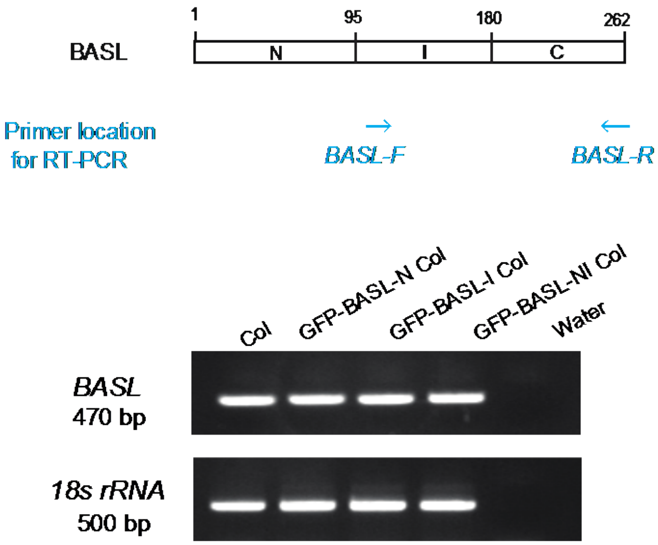

Figure S4. Semi-quantitative RT-PCR for the endogenous expression of *BASL* in the overexpression lines of BASL subdomains.

(Top panel) Primers used for evaluating endogenous BASL expression.

(Bottom panel) RT-PCR data for endogenous *BASL* expression in designated transgenic plants. The negative control (water) used ddH_2O instead of cDNA as a template and the positive control (Col) amplified *BASL* transcripts in the WT plants. Total RNAs were isolated from 7-dpg seedlings and the number of thermo cycles were 35 for *BASL* and 25 for *18s rRNA*, respectively.

Supplementary Table S1. Primers used in this study

**BASL_I2-F**	5'-CACC ATG AAG AAA GAG ACG GAA TCG TCC -3’
**BASL_I2-R**	5'- TGA GTG ACT AGA CCC GCT AGA TTT ATC -3'
**BASL-F**	5'- CACC ATG GCT TCA CAG TGG ACA ATA C-3'
**Myr-BASL_N-F**	5'- CACC ATG GGC AAC AAA TGT TGC AGC AAG CGA CAG GAT ACC ATG GCC GCA GCT ATG GCT TCA CAG TGG ACA ATA C-3'
**Myr-BASL_I-F**	5'-CACC ATG GGC AAC AAA TGT TGC AGC AAG CGA CAG GAT ACC ATG GCC GCA GCT CAG CAC CGG CGC TTG AGC TG -3'
**Myr-BASL_C-F**	5'- CACC ATG GGC AAC AAA TGT TGC AGC AAG CGA CAG GAT ACC ATG GCC GCA GCT AAC ACT TCA TGT CAA GGA AC -3'
**NLSd-F**	5'- CAC TAC GAA CAA CAA TAA TCT AAC GTT CTC AGA GAG GAA ACT CAG CCT A -3'
**NLSd-R**	5'- GAA CGT TAG ATT ATT GTT GTT CGT AGT G -3'
**NESd-F**	5'- G AGG AAG ATT AAA AGT ACT AAG AAG AAG AGA AGC CCA CCA GGT ACA CGT CAT CAT -3'
**NESd-R**	5'- ATG ATG ACG TGT ACC TGG TGG GCT TCT CTT CTT CTT AGT ACT TTT AAT CTT CCT C -3'
**NLS/NESd-F**	5'- C ACT ACG AAC AAC AAT AAT CTA ACG TTC AGC CCA CCA GGT ACA CGT CAT CAT-3'
**NLS/NESd-R**	5'- ATG ATG ACG TGT ACC TGG TGG GCT GAA CGT TAG ATT ATT GTT GTT CGT AGT G -3'
**BASL_d41-R**	5'- TCC CCT TCC TTC ATC TGA GTG -3'
**BASL_d32-R**	5'- TAC TCC TAA TAT GGG AAA TGC AAA AG -3'
**BASL_d6-R**	5'- CCC TAA AGC AAC TGG CTT TTG -3'
**BASL_AAA-F**	5'- GAA GGA AGG GGA TCT GCT GCA GCT CCC ATA TTA GGA GTA GAG-3'
**BASL_AAA-R**	5'- CTC TAC TCC TAA TAT GGG AGC TGC AGC AGA TCC CCT TCC TTC-3'
**BASL_2C2S-R**	5'- GAA TCT ACT ACT TTG GAA CCC TAA AGC AAC TGG C -3'
**BASL-F (RT-PCR)**	5’-CTGTCTCAGAAGAATCTGGATTC-3’
**BASL-R (RT-PCR)**	5’-GAATCTACAACATTGGAACCC-3’
**18s rRNA-F**	5’- CCAGCGATCGTTTATTGCTT -3’
**18s rRNA-R**	5’ AGTCTTTCCTCTGCGACCAG-3’

Supplementary Table S2. Localization and overexpression phenotype of BASL subdomain in the wild-type Col plants.

	**Nucleus**	**Cytoplasm/PM**	**PM polarization**	**Stomatal Phenotype[a]**
BASL_N	+++	+	-	-
BASL_I	+	+++	-	DN
BASL_I2	+	+++	-	-
BASL_C	+	+++	-	-
BASL_NI	+++	++	+/-	DN
BASL_IC	+	++	+++	-
Myr-BASL	-	+++	++	-
Myr-BASL_N	+	++	-	-
Myr-BASL_I	-	++	-	DN
Myr-BASL_C	-	++	-	-

[a] -, wild-type phenotype; DN, dominant negative
